# The Effect of Automated Verbal Commands During Avalanche Transceiver Search on Acute Mental Stress and Arousal—A Mixed‐Methods Crossover Field Study

**DOI:** 10.1002/brb3.70684

**Published:** 2025-07-21

**Authors:** Bernd Wallner, Fabio Caramazza, Simon Woyke, Manuel Winkler, Ivo B. Regli, Peter Mair, Gabriel Putzer, Giacomo Strapazzon, Markus Falk, Hermann Brugger, Katharina Hüfner

**Affiliations:** ^1^ Department of Anaesthesiology and Intensive Care Medicine, Innsbruck Medical University Hospital Medical University of Innsbruck Innsbruck Austria; ^2^ Institute of Mountain Emergency Medicine Eurac Research Bolzano Italy; ^3^ Department of Cardiac Anesthesiology and Intensive Care Medicine Deutsches Herzzentrum der Charité (DHZC) Berlin Germany; ^4^ eScience Bruneck Italy; ^5^ Department of Psychiatry, Psychotherapy, Psychosomatics, and Medical Psychology, University Clinic for Psychiatry II Innsbruck Medical University Innsbruck Austria

**Keywords:** avalanche medicine, companion rescue, training, transceiver search, verbal commands

## Abstract

**Background:**

Companion rescue using avalanche transceivers can lead to a disproportionate increase in mental stress and arousal, which can negatively affect performance. The aim of this mixed‐methods field study was to assess the effects of automated verbal commands on mental stress and affective responses.

**Methods:**

Participants performed two search trials using avalanche transceivers with either verbal commands (VOICE) or without verbal commands (NO VOICE) in simulated avalanche rescue scenarios. The study assessed perceived mental stress with a visual analogue scale (VAS 1–10 points) and used the Self‐Assessment‐Manikin with 9‐point Likert scales to measure affective response (valence, arousal, and dominance). Semistructured interviews were performed to assess the specific effects of verbal commands.

**Results:**

Participants reported higher levels of mental stress after the trials than before the trials (−1.2 ± 2.3 points; *p* ≤ 0.001). A smaller increase (59.0 ± 8.2 s vs. larger increase [81.0 ± 11.5 s]) in mental stress, as detected in participants using VOICE navigation, resulted in shorter coarse search times. VOICE (Δ −1.2 ± 3.2 vs. Δ −0.6 ± 2.7 points; *p* = 0.041) showed a greater reduction in arousal, resulting in faster coarse search. In the qualitative interviews, the majority described VOICE navigation to be helpful. In the individual semistructured interviews, those subjects reporting a stress reduction through VOICE (38.5 ± 4.7 s vs. no stress reduction [107.4 ± 24.3 s]; *p* = 0.001) had a significantly faster coarse search time, and those who indicated VOICE to be helpful were faster in the coarse search (*p* = 0.013).

**Conclusions:**

The study demonstrated that even a simulated avalanche rescue scenario results in increased mental stress levels. VOICE navigation may be an interesting option for companion avalanche rescue since it helps to reduce mental stress and arousal, thereby leading to shorter search times. The most significant limitation of the study was the setting of an experimentally created avalanche scenario, which cannot fully replicate all the environmental and psychological factors of a veritable burial situation.

## Graphical abstract

Avalanche search results in an increase in mental stress with longer coarse search times. VOICE navigation may be an interesting option for companion avalanche rescue since it helps to reduce mental stress and arousal, thereby leading to shorter search times.

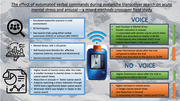

**Abbreviations**
AED: automated external defibrillator
CPR: cardiopulmonary resuscitation
PTSD: posttraumatic stress disorder
SAM: Self‐Assessment Manikin
VAS: visual analogue scale

## 1 Background

An ever‐growing number of people are indulging in sports in the mountains and in the wilderness environment, a trend that was accelerated by the COVID‐19 pandemic (Hedenborg et al. 2024, Beery et al. 2021). In contrast to indoor sport activities, outdoor sport activities tended to increase after the pandemic (Beery et al. 2021, Lee et al. 2022). In particular, backcountry skiing, ski touring, and snowshoeing on mountainous terrain have become more popular in recent years (Procter et al. 2014). Physical activity in an alpine environment has a positive effect on mental and physical health (Ower et al. 2019). Studies have shown that even viewing an alpine environment already positively affects emotional analytics in patients with somatoform, depressive, and anxiety disorders as well as in healthy controls (Hufner et al. 2020). Despite this beneficial effect, winter sports off secured ski slopes are at significant risk for avalanches.

In the case of critical burial, that is, at least involving the head and chest, the chance of survival is highly time dependent (Falk et al. 1994, Procter et al. 2016). In the initial phase, when survival rates are the highest, the companions or bystanders initiate the search and rescue operation. Companion rescue comprises searching for the victim by using an avalanche transceiver, probing, and extracting the victim from the avalanche debris (Van Tilburg et al. 2024). Unfortunately, not all mountaineers are sufficiently trained in avalanche rescue (Procter et al. 2014), which can then lead to a disproportionate increase in mental stress and arousal, which in turn can negatively affect search performance (Rahman et al. 2012). High arousal in a traumatic situation is also a risk factor for posttraumatic stress disorder (PTSD) (Finnsdottir and Elklit 2002, Leonard et al. 2021, Salvotti et al. 2024). Recently, transceivers with automated verbal commands have been introduced to help the user locate a critically buried avalanche victim in the shortest possible time. The aim of the current mixed‐methods field study was to assess the effect of automated verbal commands during avalanche transceiver search on measures of mental stress and affective response valence, arousal, and dominance.

## 2 Materials and Methods

This prospective mixed‐methods crossover study investigated the impact of a new voice navigation technology and the effect of automated verbal commands on mental stress and affective responses during the course of an avalanche transceiver search in a simulated avalanche scenario. This was part of a larger study on the effect of verbal commands on avalanche rescue. The ethics committee of the Medical University of Innsbruck, Austria, was contacted and considered a rigorous ethical evaluation unnecessary (inquiry to the Ethics Committee on December 23, 2021). After being informed in detail about the study aims and procedures, all participants provided informed consent prior to study participation. The study was conducted over 2 days in March 2022.

### 2.1 Participants and Study Site

Participants over 18 years of age performing winter sports in a recreational manner with no or very limited experience in avalanche search and no previous experience with avalanche transceivers were included in the study. Included participants had no previous experience with similar VOICE navigation technologies. Participants who reported any form of physical or mental disorders, pregnancy, or regular medication were excluded.

The study site was located at 2450 m asl near the skiing area Kühtai in Tyrol, Austria. The objective environmental parameters during the course of the experiments were the meteorological conditions (mostly sunny, temperature during the experiment 2°C–7°C; hardly any wind), high altitude, and physical exertion. The condition of the snow (the snow density had a median of 320 kg/m^3^) can be compared to the avalanche debris.

An avalanche field was prepared on a level surface with two transmitters buried at a depth of 100 cm under the snow surface. Both transmitters were placed exactly at a distance of 18 m from the starting point, which was located right of the center between both transmitters. Transmitters were attached to wood panels coated with elastic material to mimic an avalanche victim's trunk. During the trials, the weather was sunny with clear visibility. The avalanche transceiver “diract voice” (Ortovox, diract voice avalanche beacon, Ortovox Sportartikel GmbH, Taufkirchen, Germany; https://www.ispo.com/en/promotion‐ortovox/ortovox‐avalanche‐transceiver‐voice‐navigation‐diract‐voice) was used for randomization with or without voice navigation. A conventional avalanche probe (Ortovox Sportartikel GmbH) was used.

### 2.2 Study Protocol

One week prior to the trials, a video with structured instructions on how to perform an avalanche search and how to adequately use a standard avalanche transceiver (not the ones used in the experiment) and probe was sent to each participant. Immediately prior to each trial, the participants were confronted with the hypothetical scenario of being part of a skiing team, and one of their teammates was buried by an avalanche to simulate a realistic scenario. All participating researchers were instructed to only interact with participants in a very professional and distant manner and to refrain from any encouragement or small talk with the participants in order not to influence stress levels through human interaction.

**FIGURE 1:**
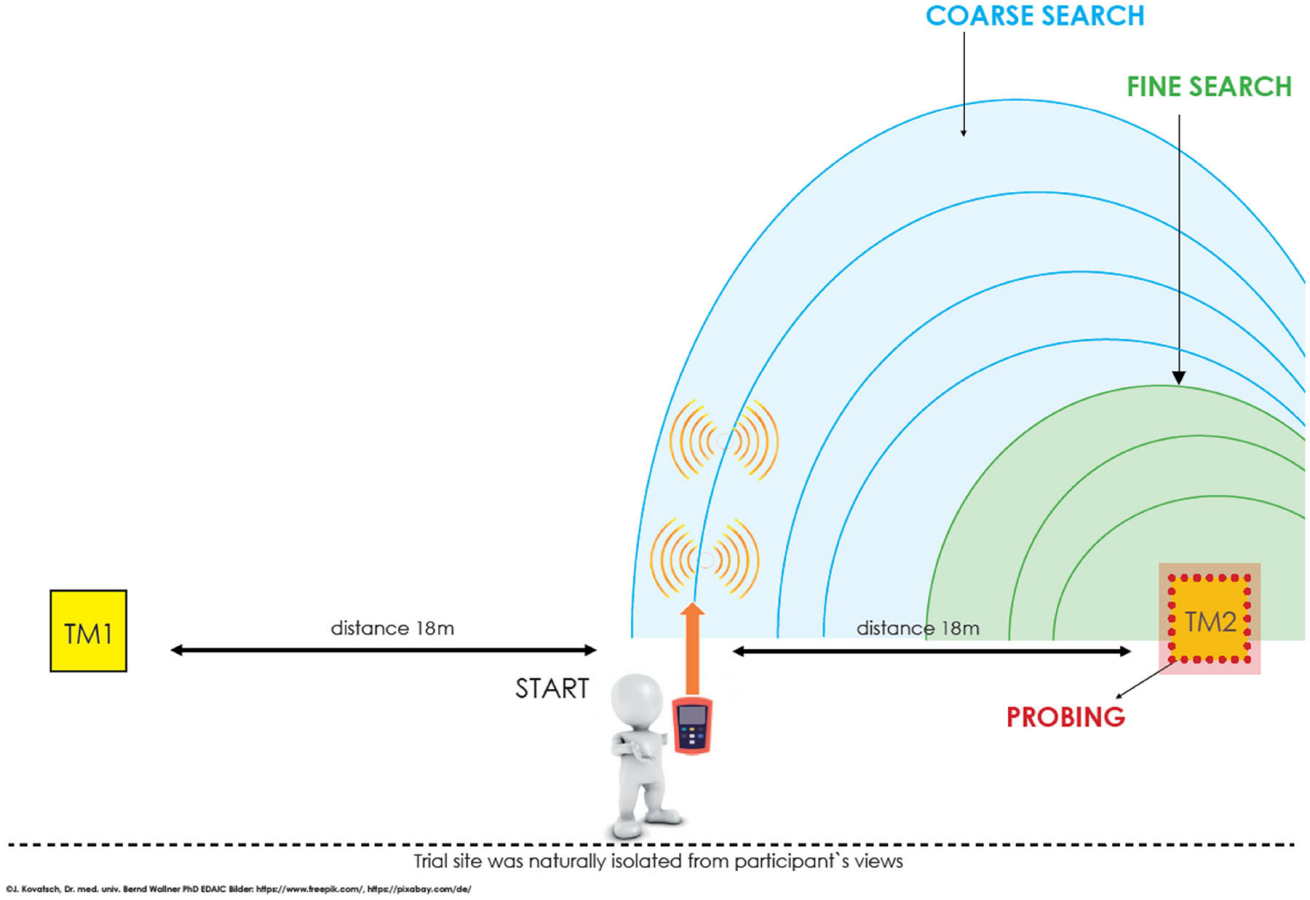
Graphical display of the trial site. Participants always started at the same spot. Either transmitter TM1 or TM2 was randomly activated. Participants started each trial in a coarse search (blue area) before they reached a fine search and then switched to probing.

Each participant performed two trials, and the activation of voice navigation by the avalanche transceiver and the choice of transmitters (left or right, TM1 or TM2; see Figure 1) were randomized. The trials were complete when the participant hit the transmitter panel with the probe and called out to have a “hit.” The trial was stopped after 10 min or three incorrect “hit” callouts by the participants.

### 2.3 Measurements and Psychometric Tests

Several time points were observed: the first time interval (coarse search) was defined as the time from the start of the trial to the beginning of the fine search; the second time interval (fine search) was defined as the time from the beginning of the fine search until the participant started using the probe; and the third time interval (probing) was defined as the time from the start of probing to trial completion.

Sociodemographic parameters included information on age, sex, and the type and frequency of winter sports the participant was performing (e.g., ski, snowboarding, ski touring, snowshoeing). Further questions asked included how often the participant was practicing the sport in the mountains in backcountry areas, how often the participant attended an avalanche course, how often the participant used an avalanche transceiver or a probe, and whether the participant had ever observed an avalanche.

#### 2.3.1 Arousal, Valence, and Dominance Self‐Ratings (Self‐Assessment Manikin [SAM] Ratings)

To measure affective responses, we used the SAM with 9‐point Likert scales. This scale measures emotional analytics in three dimensions: arousal, valence, and dominance (Branco et al. 2023, Libkuman et al. 2007, Mikels et al. 2005). The original examples of the SAM can be found in the publication by Bradley and Lang (1994).

The arousal scale displays the lowest value with a calm, eyes‐closed figure (relaxed, calm, sluggish, dull, sleepy, unaroused), while the highest value is represented by an excited figure (stimulated, excited, frenzied, jittery, wide‐awake, aroused). The valence scale ranges from a frowning, unhappy (adjectives used in the SAM manual: unhappy, annoyed, unsatisfied, melancholic, despaired, bored, lower values) to a smiling, happy figure (happy, pleased, satisfied, contented, hopeful). The lowest values in the dominance scale are symbolized by a controlled small figure (controlled, influenced, cared for, awed, submissive, and guided), while the highest values are represented by a dominant and oversized figure (controlling, influential, in control, important, dominant, autonomous). The challenges of such assessment tools are well known in previous studies, and additional tools are being investigated (Diotaiuti et al. 2020).

#### 2.3.2 Self‐Rating of Mental Stress

Perceived mental stress was assessed using a visual analogue scale (VAS) ranging from 1 to 10 points, with 1 indicating “no stress at all” and 10 indicating “the highest level of stress possible.” In the statistical analysis, we calculated the difference in stress level POST (after each trial) minus PRE (prior to each trial). A positive result indicated an increase in the stress level, and a negative result indicated a decrease in the stress level during the trial.

#### 2.3.3 Qualitative Data Collection

A personal, semistructured interview was performed with each participant after the completion of both trials by authors F.C. and K.H. The questions asked can be found in the Supporting Information. The participants were asked about the detailed aspects of the effects that the VOICE device had on the perceived levels of mental stress, arousal, and performance.

### 2.4 Statistical Analysis

Data from a pilot study revealed an average search time of 120 s with a standard deviation of approximately 60 s. To detect a clinically relevant difference of 20 s between methods, with two repetitions per subject, a sample size of 50 subjects would be necessary to achieve 80% power, with an alpha level of 5%. Group mean differences were evaluated using the *t*‐test, while pre‐ versus post‐comparisons employed the paired *t*‐test. Group comparisons of counted data were conducted using the chi‐square test, while time to rescue was analyzed using the Kaplan‒Meier estimator, with group comparisons performed via the log‐rank test. Continuous data are presented as the mean and standard deviation or as the median with range, as appropriate, while frequencies were utilized for counted data. Statistical analyses were carried out using SPSS 29, with significance set at a two‐sided *p*‐value less than 0.05. In addition, the correlation was calculated using Pearson correlation. As we also have to take censoring into account for the times, we therefore also validated the correlation using a Cox model.

## 3 Results

The mean age of the 50 volunteers (50% female and 50% male) was 24.0 ± 3.6 years. All participants were medical students at the Medical University Innsbruck, Austria. None of the participants reported any previous experience using an avalanche transceiver or knowledge of avalanche rescue techniques. Ninety‐five of 100 individual trials were completed, and five trials were terminated by the experimenters because the predefined termination criteria were met: in two trials, the maximum trial time of 10 min was exceeded (one using VOICE, one NO VOICE), and three trials were terminated because of probing failure after three erroneous hits (one using VOICE, two NO VOICE).

### 3.1 Mental Stress

Participants reported higher levels of mental stress after the trial than before the trial (*p* ≤ 0.001) (paired *t*‐test; mean diff. −1.2 ± 2.3; *p* ≤ 0.001; |*d*| = −0.52) overall and when analyzing the use of VOICE (*p* = 0.006) (paired *t*‐test; mean diff. −1.0 ± 2.5; *p* ≤ 0.006; |*d*| = −0.41) and NO VOICE (paired *t*‐test mean diff. −1.4 ± 2.1; *p* ≤ 0.001; |*d*| = −0.66) for navigation (Table 1). NO VOICE (Δ1.4 points) navigation showed a greater but not significantly greater increase in stress level than did VOICE navigation (Δ1.0 points, *p* ≤ 0.329). When mental stress levels were directly compared between conditions (VOICE vs. NO VOICE), there was no significant difference either before or after each trial.

**TABLE 1:** Mental stress, arousal, and dominance before (PRE) and after (POST) each trial, as well as the difference in the POST–PRE score as a function of the mean and *SD*. All three parameters were assessed using a visual analogue scale (VAS) ranging from 1 to 10 points, with 1 indicating “no stress at all” and 10 indicating “the highest level of stress possible.”

Mental stress, arousal, and dominance ‐ Paired samples test							
	Paired differences (mean)	PRE	POST	POST − PRE (Mean ± *SD*)	*T*‐test	Cohen's *d*
Mental stress	VOICE	5.9	7.0	1.0 ± 2.5	*p* = 0.006	−1.3
	NO VOICE	6.0	7.3	1.4 ± 2.1	*p* < 0.001	−1.9
	Δ NO VOICE – VOICE			0.5 ± 3.5	*p* = 0.329	0.5
Arousal	VOICE	2.4	1.2	−1.2 ± 3.2	*p* < 0.001	1.9
	NO VOICE	2.1	1.5	−0.6 ± 2.2	*p* = 0.009	1.0
	Δ NO VOICE – VOICE			−0.6 ± 2.2	*p* = 0.041	−0.8
Dominance	VOICE	4.0	4.2	−0.2 ± 1.4	*p* = 0.382	−0.3
	NO VOICE	3.6	4.3	0.7 ± 2.7	*p* = 0.017	−1.0
	Δ NO VOICE – VOICE			0.5 ± 2.9	*p* = 0.207	0.6

**FIGURE 2:**
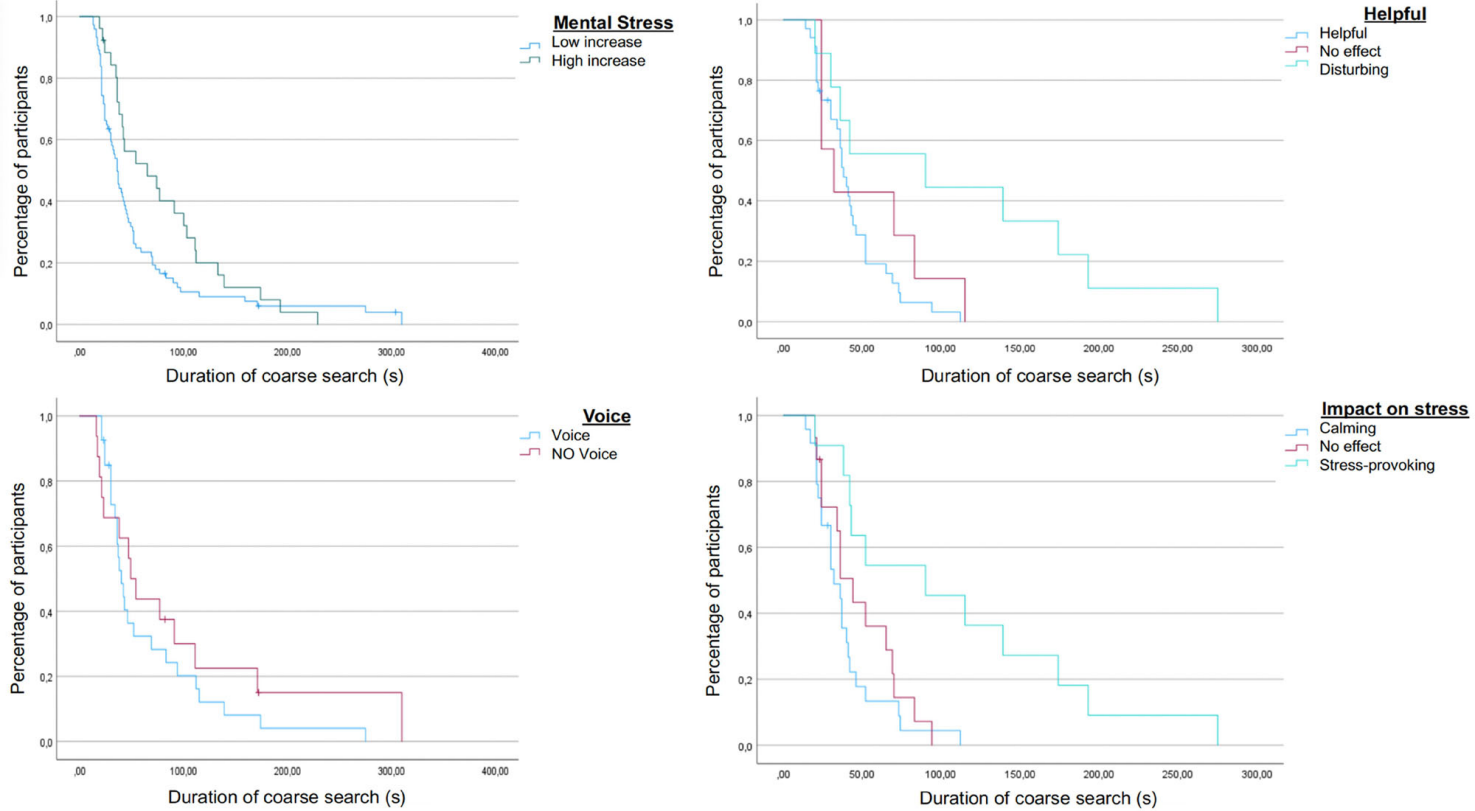
Comparison between a high increase and a low increase in mental stress on the duration of the coarse search in seconds (top left). The effect of the perception of VOICE navigation (helpful, disturbing, no effect/neutral) on the duration of the coarse search in seconds. When VOICE navigation was perceived as disturbing, this resulted in a significant increase in the duration of the coarse search (top right). In participants with an increased arousal, the use of VOICE navigation resulted in shorter coarse search time in seconds (bottom left). The influence of stress and the effect of VOICE navigation on the duration of the coarse search. In participants where VOICE navigation had a calming effect, a significantly shorter coarse rough search was observed (bottom right).

The median increase in mental stress level was 2.00 (= median difference POST − PRE). A large increase in mental stress level was defined as >2.00, and a small increase in mental stress level was defined as ≤2.00. An increase in mental stress had a significant impact on the duration of the coarse search (*p* = 0.046). Those participants reporting a small increase in mental stress (*n* = 74 of 100 trials) were faster in the coarse search, leading to shorter coarse search times (small increases in mental stress mean 59.0 ± 8.2 s; 95% CI 43.0–74.9) (Figure 2). Those participants who reported a large increase in mental stress (*n* = 26 of 100 trials) were slower in the coarse search (81.0 ± 11.5 s; 95% CI 58.6–103.5; *p* = 0.046). A further analysis of the correlation showed a direct correlation between mental stress and certain search times. A reduction in mental stress correlated with a decrease in the fine search (Cox model *p* = 0.005), in the probing time (Cox model *p* = 0.037), and in the total search time (Cox model *p* = 0.018).

### 3.2 Arousal

In 43% of trials, participants reported an increase and, in 57%, a decrease in arousal following the search compared to prior to the search. Overall, the mean arousal value was lower following the trial than before the trial (*p* ≤ 0.001) (paired *t*‐test; mean diff. −0.9 ± 1.6; *p* ≤ 0.001; |*d*| = 0.56) (Table 1). Lower levels of arousal after the trial were also identified when analyzing the two conditions separately (VOICE, *p* ≤ 0.001) (paired *t*‐test; mean diff. −1.2 ± 1.7; |*d*| = 0.74) and NO VOICE (*p* = 0.009) (paired *t*‐test; mean diff. −0.6 ± 1.5; *p* = 0.009; |*d*| = 0.39) (Table 1). Compared with the condition in which VOICE navigation was not used, the condition in which VOICE navigation was used showed a greater reduction in arousal (*p* = 0.041) (paired *t*‐test; mean diff. at an arousal reduction of −0.6 ± 2.2; *p* = 0.041; |*d*| = 0.30) (Table 1). The analysis of the impact of arousal on the search times of the participants showed that those with a decrease in arousal (after each trial compared to before) were faster than participants who did not show this effect (mean 54.8 ± 7.2 s vs. 76.3 ± 12.0 s, *p* = 0.045) (Figure 2). In participants with increased arousal after the search, VOICE was associated with faster search times (*p* = 0.026) (mean 66.3 ± 11.9 s vs. 97.0 ± 26.5 s; CI 42.9–89.6; Pearson chi‐square test).

### 3.3 Dominance

The only significant difference in dominance values was found in those trials where NO VOICE was used. Participants using NO VOICE reported greater dominance values after the trial than before the trial (*p* = 0.017) (paired *t*‐test; mean diff. −0.7 ± 2.1; *p* = 0.017; |*d*| = −0.97) (Table 1). This effect was not present in the trials in which the VOICE navigation was used. A direct comparison of the trials using VOICE with those using NO VOICE did not reveal any significant differences.

### 3.4 Valence

Valence was the only parameter that did not differ before and after the trial (paired *t*‐test; VOICE navigation, *p* = 0.369; NO VOICE navigation, *p* = 0.526). Furthermore, the analysis of valence did not reveal a significant difference between the VOICE navigation and the NO VOICE navigation (*p* = 0.769). In the calculation of the correlation, a direct correlation between valence and mental stress was demonstrated (*p* < 0.001).

### 3.5 Results From the Individual Semistructured Interviews

In the individual semistructured interviews, 68.0% of the participants described VOICE navigation to be helpful, 18% found it to be disturbing, and 14% reported no effect (neutral). The helpful effect of VOICE navigation was associated with a reduction in stress level (87.5%, *p* = 0.002). Participants based the helpful effect of VOICE navigation mainly on the reason that VOICE navigation conveyed a sense of security (*p* = 0.084), provided a correction of mistakes (*p* = 0.014) and confirmation of performed actions (*p* = 0.014). Further associations of the helpful effect of VOICE navigation included an increased level of concentration (*p* = 0.045) and an absence of confusion (*p* = 0.002). VOICE navigation, however, did not significantly increase the level of motivation. VOICE navigation was further regarded as helpful because it provided instructions with otherwise missing prior background knowledge (*p* = 0.027).

The effect of verbal commands on the perceived levels of mental stress was further specified in a semistructured interview. Those subjects (*n* = 27 of 50 participants) who indicated a stress reduction through the calming effect of VOICE, in turn, had a significantly faster coarse search time (38.5 vs. 107.4 s) (*p* = 0.001) than did those who reported an stress‐provoking effect (*n* = 8 of 50 participants) of VOICE (calming effect mean 38.5 ± 4.7 s; 95% CI 29.3–47.7; stress‐provoking effect mean 107.4 ± 24.3 s; 95% CI 59.7–155.1; *p* = 0.001 log rank test) (Table 2). When analyzing the performance and the search times in relation to the effect of VOICE, the data showed that those who indicated VOICE navigation to be helpful were faster in the coarse search (helpful mean 42.9 ± 4.0 s; 95% CI 35.1–50.8; disturbing mean 111.0 ± 29.8 s; 95% CI 42.9–169.5; *p* = 0.013 log rank test). This effect was mainly attributed to an increased level of concentration (*p* = 0.024) and the confirmation of performed actions (*p* = 0.037) (Figure 2).

**TABLE 2:** Log rang effects of VOICE on participants and on the duration of coarse search.

Effects of VOICE on participants’ performance				
	Coarse search (mean)	*SE*	CI (95%)	*p*‐value
Calming effect by VOICE (n = 27/50)	38.5 s	±4.7	29.3–47.7	0.001
Neutral effect by VOICE (n = 27/50)	48.3 s	±6.5	35.6–61.0	0.013
Stress‐provoking effect by VOICE (n = 8/50)	107.4 s	±24.3	59.7–155.1	0.001

## 4 Discussion

This study was the first to evaluate the impact of a search operation after a simulated avalanche accident on psychological parameters, namely, mental stress and affective responses. The study further analyzed the effect of verbal commands from a new generation of avalanche transceivers on those parameters and the performance of laypersons.

The most important findings of our study were as follows:

VOICE and NO VOICE showed an increase in mental stress, which was less pronounced when using VOICE navigation. Greater increases in mental stress were associated with longer coarse search times.

Both VOICE and NO VOICE showed a reduction in arousal following the trial, with a significantly greater reduction using VOICE navigation. Individuals who showed a decrease in arousal (after each trial compared to before) were faster in the coarse search than participants who did not show this effect. In the subgroup of participants who reported an increase in arousal following the search compared to prior to the search, the use of VOICE was associated with faster search times.

Participants using NO VOICE navigation reported significantly greater dominance after the trial than immediately prior to the trial.

In the individual semistructured interviews, more than two thirds of all participants described VOICE navigation to be helpful.

The participants who indicated that VOICE navigation was helpful in the interviews were faster in the coarse search, which they attributed to an improvement in concentration and a reduction in the perceived stress level.

Verbal commands are increasingly being used in medical devices. Automated external defibrillators (AEDs) are increasingly fitted with verbal commands and provide an impressive example of enhancing user interactions with a medical device. The latest‐generation AEDs make use of verbal commands to guide CPR (cardiopulmonary resuscitation) providers through the required steps while performing successful CPR (Beckers et al. 2005). In contrast, verbal commands that do not transport an adequate amount of information or distract from the actual task could create confusion. Clearly, the use of voice prompts in an AED has been shown to be both helpful and reassuring during a stressful event (Beckers et al. 2005).

The mental stress observed in the participants was significantly greater after each test run than before. The effect may be explained by the nature of the simulated situation. The study team aimed to create an atmosphere that re‐enacted the stressful situation after an avalanche accident. In addition to their immense responsibility, which was transferred to the participants, they had to run over avalanche debris, which is a strenuous physical exercise to be performed at altitude (2.450 m asl).

This increase in stress was less pronounced in the participants who used VOICE navigation. When analyzing the performance of the participants in each trial, we found that those participants who had lower levels of stress performed better and had shorter coarse search times. This finding is in accordance with various studies found in the literature. One study revealed that acute psychological stress provocation led to decreased performance in elite male swimmers and that prerace cortisol levels were positively associated with lactate (Rano et al. 2019). In a different study, floorball players’ physical and psychosocial stress impacted performance (van der Does et al. 2015). The correlation analysis of mental stress and search times shows a clear linear correlation. An increase in mental stress was associated with an increase in valence and significantly longer search times. The clearest correlation was shown in an increase in those times when focusing and concentrating on accuracy and precise work were of the greatest importance, namely, in the fine search and during probing.

We found that individuals who described VOICE navigation as helpful or calming reported lower levels of stress and were significantly faster in the coarse search. One possible explanation may be the fact that certain participants use VOICE navigation and utilize the device for their benefit. Other participants found it difficult to accept the information and described the VOICE navigation to be distracting. This finding is in accordance with other studies that state that in certain cases, verbal commands may cause confusion. A study analyzing the performance of laypersons operating AEDs concluded that simple devices should be developed with clear instructions, and it reiterates that design, construction, and visual aids have an impact on user performance (Eames et al. 2003). This statement was confirmed by the observation of Beckers et al. that even in the second evaluation, in the automatic group (including voice prompts), three students were unable to deliver a shock (Beckers et al. 2005).

The present results have additional implication for the design of future devices being used in search and rescue devices as well as human–machine interfaces (HMIs). One study on the development of HMI in disaster‐purposed search robot systems found that these systems aim to extend the existing robotics and human interface technologies and to establish a novel human–machine interacting structure to enable effective use of semiautonomous robots in place of humans for searching survivors from rubble under human remote instructions (Zheng et al. 2004).

In contrast to an increase in mental stress, the analysis of arousal showed a different picture. The majority of participants (57%) reported a lower level of arousal following the trial than directly prior to the test. Nevertheless, 43% of participants described an increase in arousal. When the two conditions, VOICE and NO VOICE navigation, were analyzed separately, a significant overall reduction in arousal levels was identified in favor of VOICE navigation. The condition using VOICE navigation showed a significantly greater reduction in arousal than did the condition using NO VOICE. As previously described, humans experience emotional arousal when threats to life and limb are imminent, such as in high stakes and emergency situations that occur in sociotechnical systems such as firefighting, aviation, and combat (Rahman et al. 2012). Emotional arousal has both advantages and disadvantages. On the downside, it introduces perceptual distortions and biases and an inability to process symbolic information and alters motor abilities. For this purpose, various emergency HMIs have been designed to accommodate the human capacities that have been altered by danger‐induced emotional arousal.

Another highly interesting aspect is the course of arousal during the trial. We observed a decrease in arousal sometime after the trial, during a period when mental stress levels were still high. One possible explanation might be the different changes in arousal and mental stress over time. The authors hypothesize that arousal decreases faster immediately after the trial, while mental stress persists at a higher level and decreases only at a later stage. This later decrease was then detected in the semistructured interviews where participants reported a stress reduction when using VOICE, which could not be found in the quantitative tests. This finding has already been described in a study that showed that subjective stress perception was significantly greater immediately after the acute stressor than before it and started to decrease only 20 min after the acute stressor (Becker and Rohleder 2019).

The only significant effect of dominance was observed using NO VOICE navigation, where participants reported higher levels of dominance after the test trial. This result shows that dominance is closely related to self‐control and self‐efficacy. Higher dominance ratings could indicate that the rescuer feels more in control. This can be beneficial for decision‐making in difficult situations. A recent study analyzed the integration of self‐efficacy and response efficacy in decision‐making and found that their interaction in shaping individuals’ decisions can enhance our comprehension of how perceived control influences choices, coping strategies, and overall psychological health (Yang and Delgado 2025). Participants who did not receive any help in the form of voice commands stayed in control and had to determine more by themselves. Valence does not seem to be influenced by VOICE navigation or by the order of the test trials, and since valence is an inferred criterion from instinctively generated emotions, it can be deduced that the entire trial does not influence the extent to which emotions are pleasant or unpleasant.

One possible consequence of being involved in an avalanche rescue operation is the subsequent development of PTSD not only in possible victims but also in the rescuers. High levels of mental stress and increased arousal have been found to possibly be risk factors for the development of PTSD; therefore, addressing affective responses and mental stress during avalanche rescue could also have long‐term positive health consequences (Salvotti et al. 2024, Sareen 2014, van der Kolk 2000, Tian et al. 2014). Stress and arousal reduction are among the few factors that predict the occurrence of PTSD.

### 4.1 Limitations of the Study

Although the present study was conducted off secured slopes, there are still many factors that cannot be included in this standardized experiment. We do not know what happens when other stressors, such as cold or fatigue, are added. We also concede that although the investigators tried to build up a certain level of stress, it is not possible to achieve the same level of stress as in an actual avalanche burial.

The present study was carried out on participants without any previous experience; therefore, no statements can be made about people with a certain amount of previous experience. The current recommendations are very much in favor of regular training in the search for avalanche victims; therefore, it can be assumed that most athletes will have at least minimal knowledge of avalanche burial search and the results of this study cannot be generalized to athletes with more experience.

For certain participants, the mental stress scale differed substantially from that used in the individual interviews. This might be because the mental stress scale was evaluated immediately, and the interview was conducted at the end of both test trials, when most of the stress had already decreased and the tension had been removed.

## 5 Conclusion

This was the first study to evaluate the impact of search and rescue operation after a simulated avalanche accident on psychological parameters and discovered that the performance of a simulated avalanche transceiver search resulted in an increase in mental stress but a reduction in arousal. Greater increases in mental stress were associated with longer coarse search times. Individuals who showed a decrease in arousal (after each trial compared to before) were faster in the coarse search than participants who did not show this effect. In the subgroup of participants who reported an increase in arousal following the search compared to prior to the search, the use of VOICE navigation was associated with faster search times. The majority of participants rated VOICE navigation as helpful, and those who indicated VOICE navigation to be helpful in the interview were faster in the coarse search, which they attributed to an improvement in concentration and a reduction in the stress level. For future studies and more in‐depth insights, it is recommended to include additional control groups and longitudinal follow‐ups, as well as addition analysis to further clarify the causal pathways.

## Author Contributions

**Bernd Wallner**: conceptualization, investigation, writing – original draft, methodology, validation, visualization, writing – review and editing, data curation, resources. **Fabio Caramazza**: conceptualization, investigation, writing – original draft, writing – review and editing. **Simon Woyke**: conceptualization, investigation, writing – original draft, writing – review and editing, methodology. **Manuel Winkler**: conceptualization, investigation, writing – original draft, writing – review and editing, project administration. **Ivo B. Regli**: conceptualization, investigation, writing – original draft, writing – review and editing. **Peter Mair**: conceptualization, investigation, writing – original draft, writing – review and editing, validation, supervision, project administration. **Gabriel Putzer**: conceptualization, investigation, writing – original draft, writing – review and editing. **Giacomo Strapazzon**: conceptualization, investigation, writing – original draft, writing – review and editing, project administration, supervision. **Markus Falk**: conceptualization, investigation, writing – original draft, writing – review and editing, software, formal analysis, methodology, data curation. **Hermann Brugger**: conceptualization, investigation, writing – original draft, writing – review and editing, project administration, supervision, resources, validation, methodology. **Katharina Hüfner**: conceptualization, investigation, writing – original draft, methodology, validation, writing – review and editing, project administration, supervision, data curation.

## Ethics Statement

The ethical committee of the Medical University Innsbruck was contacted, and the patients were informed about the study. The need for ethical approval was waived. All participants provided written informed consent for their participation in the study and for the publication of the data.

## Consent

All participants provided written informed consent for the publication of the data.

## Conflicts of Interest

The company Ortovox supported this study with logistical support and provided the avalanche transceivers and the transmitters buried in the snow as well as catering to the participants of the study. A collaborator of Ortovox assisted in the planning and the implementation of the study. No financial support was provided, and none of the coauthors received any form of nonfinancial compensation for their work.

## Peer Review

The peer review history for this article is available at https://publons.com/publon/10.1002/brb3.70684

## Supporting information




**Supplemental Information Appendix**: brb370684‐sup‐0001‐SuppMat.docx

## Data Availability

The datasets used and analyzed during the current study are available from the corresponding author upon reasonable request.
