# Supplementary material for: The Effect of Automated Verbal Commands During Avalanche Transceiver Search on Acute Mental Stress and Arousal—A Mixed‐Methods Crossover Field Study
Source: Brain Behav. 2025 Jul 21;15(7):e70684. doi: 10.1002/brb3.70684 (PMC12277655; doi:10.1002/brb3.70684)
Supplement: Supplementary file 1 — Supplemental Information Appendix: brb370684‐sup‐0001‐SuppMat.docx [file BRB3-15-e70684-s001.docx]

**Appendix – Supplemental information**

Qualitative Data collection

The following questions were used (only the questions in bold were asked to all participants, the other questions were asked in case participants did not spontaneously):

- **How did you find voice navigation when searching? (Helpful/without effect/disturbing)**
- Why? What was helpful/disturbing about it?
- Was the type of voice helpful/neutral/disturbing?
- Was the content of what was said helpful/disturbing?
- Was the content of what was said clear/unclear?
- Were you able to concentrate worse/better with voice navigation?
- What behaviour/feeling did the voice trigger?
- **How did voice navigation affect your mental stress level? (Calming/neutral/stress- provoking)**
- Why? What was calming/stress-provoking about the voice navigation?
- Was the type of voice calming/stress- provoking?
- Was the content of what was said calming/stress- provoking?
- What did the voice trigger in you in relation to the stressful situation?
- Any additional information given spontaneously by the participants during the interview was recorded.

Precise calibration of voice commands and the according triggers

| Voice Command | Trigger |
| --- | --- |
| *"Run in 50 meter search strips and look out"* | instruction before signal search |
| *"Run straight"* | immediately after signal detection, if signal comes from the front |
| *"Run to the right"* | immediately after signal detection, if signal comes from the right |
| *"Run to the left"* | immediately after signal detection, if signal comes from the left |
| *"Turn around"* | in case of 180° wrong initial search direction |
| *"Keep right"* | correction if direction indication is not followed |
| *"Keep left"* | correction if direction indication is not followed |
| *"Walk slowly"* | instruction before slow coarse search |
| *"Go down to the snow surface"* | instruction before fine search |
| *"Search the smallest value"* | instruction at fine search start |
| *"You were closer"* | information when distance value is clearly increasing during fine search |

Key Components of the Coding Process for the semi-structured interviews:

The objective of the interview was to get deeper insights into possible mechanisms underlying the mode of action of VOICE navigation on psychological measures. The interview contained two parts.

The first part started with the closed question: “How did you find the voice navigation during the search? Helpful, neutral, disturbing” and then went on to explore possible mechanisms of action.

The second part of the interview started with the closed question: “How did voice navigation affect your mental stress level? Calming, neutral, stress provoking” and then went on to explore passible mechanisms of action.

The following codes were identified using deductive analysis and documented in Excel®. Subcodes were implemented where appropriate. Inductive analysis was used only for the 2^nd^ order subcodes. The units of analysis were meaning units.

| Main codes | Subcodes | Subcodes 2^nd^ order |
| --- | --- | --- |
| Perception of VOICE navigation | Helpful | Did not feel left alone |
|  |  | Sense of security |
|  |  | Correction of mistakes through voice navigation |
|  |  | Motivation |
|  |  | Increased level of concentration |
|  |  | Confirmation of performed actions |
|  |  | Perceived competence |
|  |  | Guidance in absence of prior knowledge |
|  |  | Freed mental capacity for the environment |
|  |  | other |
|  | Neutral |  |
|  | Disturbing | Confusion |
|  |  | Time pressure |
|  |  | Other |
| Impact of VOICE on Stress level | Calming | Did not feel left alone |
|  |  | Sense of security |
|  |  | Correction of mistakes through voice navigation |
|  |  | Motivation |
|  |  | Increased level of concentration |
|  |  | Confirmation of performed actions |
|  |  | Perceived competence |
|  |  | Guidance in absence of prior knowledge |
|  |  | Freed mental capacity for the environment |
|  |  | other |
|  | Neutral |  |
|  | Stress-provoking | Confusion |
|  |  | Time pressure |
|  |  | Other |
| Type/Sound of VOICE | Helpful |  |
|  | Neutral |  |
|  | Disturbing |  |
| Content of VOICE navigation | Clear |  |
|  | Neutral |  |
|  | Unclear |  |
| Subjective perception of clarity and acoustics of VOICE prompts | Clear |  |
|  | Unclear |  |
|  | Quiet |  |
| Impact of VOICE navigation on the level of focus on the required task | Better |  |
|  | Neutral |  |
|  | Worse |  |

The coding process was manually done by one coder (F.C.) any open/unclear questions were resolved with a second coder (K.H.) in consensus following discussion. Analytic notes were kept in the Excel file to capture thoughts, questions, and interpretations during the coding process.
